# End of season influenza vaccine effectiveness in adults and children in the United Kingdom in 2017/18

**DOI:** 10.2807/1560-7917.ES.2019.24.31.1800488

**Published:** 2019-08-01

**Authors:** Richard Pebody, Abdelmajid Djennad, Joanna Ellis, Nick Andrews, Diogo F P Marques, Simon Cottrell, Arlene J Reynolds, Rory Gunson, Monica Galiano, Katja Hoschler, Angie Lackenby, Chris Robertson, Mark O’Doherty, Mary Sinnathamby, Nikolaos Panagiotopoulos, Ivelina Yonova, Rebecca Webb, Catherine Moore, Matthew Donati, Muhammad Sartaj, Samantha J Shepherd, Jim McMenamin, Simon de Lusignan, Maria Zambon

**Affiliations:** 1 Public Health England, United Kingdom; 2 Health Protection Scotland, Glasgow, United Kingdom; 3 Public Health Wales, Cardiff, United Kingdom; 4 West of Scotland Specialist Virology Centre, Glasgow, United Kingdom; 5 University of Strathclyde, Glasgow, United Kingdom; 6 Public Health Agency Northern Ireland, Belfast, United Kingdom; 7 University of Surrey, Guildford, United Kingdom; 8 Royal College of General Practitioners, London, United Kingdom

**Keywords:** influenza vaccine effectiveness

## Abstract

**Background:**

In the United Kingdom (UK), in recent influenza seasons, children are offered a quadrivalent live attenuated influenza vaccine (LAIV4), and eligible adults mainly trivalent inactivated vaccine (TIV).

**Aim:**

To estimate the UK end-of-season 2017/18 adjusted vaccine effectiveness (aVE) and the seroprevalence in England of antibodies against influenza viruses cultured in eggs or tissue.

**Methods:**

This observational study employed the test-negative case–control approach to estimate aVE in primary care. The population-based seroprevalence survey used residual age-stratified samples.

**Results:**

Influenza viruses A(H3N2) (particularly subgroup 3C.2a2) and B (mainly B/Yamagata/16/88-lineage, similar to the quadrivalent vaccine B-virus component but mismatched to TIV) dominated. All-age aVE was 15% (95% confidence interval (CI): −6.3 to 32) against all influenza; −16.4% (95% CI: −59.3 to 14.9) against A(H3N2); 24.7% (95% CI: 1.1 to 42.7) against B and 66.3% (95% CI: 33.4 to 82.9) against A(H1N1)pdm09. For 2–17 year olds, LAIV4 aVE was 26.9% (95% CI: −32.6 to 59.7) against all influenza; −75.5% (95% CI: −289.6 to 21) against A(H3N2); 60.8% (95% CI: 8.2 to 83.3) against B and 90.3% (95% CI: 16.4 to 98.9) against A(H1N1)pdm09. For ≥ 18 year olds, TIV aVE against influenza B was 1.9% (95% CI: −63.6 to 41.2). The 2017 seroprevalence of antibody recognising tissue-grown A(H3N2) virus was significantly lower than that recognising egg-grown virus in all groups except 15–24 year olds.

**Conclusions:**

Overall aVE was low driven by no effectiveness against A(H3N2) possibly related to vaccine virus egg-adaption and a new A(H3N2) subgroup emergence. The TIV was not effective against influenza B. LAIV4 against influenza B and A(H1N1)pdm09 was effective.

## Introduction

The United Kingdom (UK) has a long-standing selective influenza immunisation programme offering inactivated vaccine to persons ≥ 65 years of age and those aged 6 months to 64 years of age with an underlying clinical risk factor. Following advice from the Joint Committee of Vaccination and Immunisation (JCVI), the UK started a phased introduction of a universal childhood influenza vaccine programme in 2013/14 [1]. By 2017/18, all children 2–8 years of age across the UK were being offered quadrivalent live attenuated influenza vaccine (LAIV4), with uptake higher than the previous season in targeted cohorts [2]. In addition, in England, besides children 2–8 years of age, all remaining children of primary school age (9–11 years of age) in discrete geographical pilots were offered LAIV4. Scotland and Northern Ireland also offered LAIV4 to all children of primary school age (including 9–11 years of age). A B/Yamagata lineage virus (B/Phuket/3073/2013-like virus) and a B/Victoria lineage virus (B/Brisbane/60/2008-like virus) were contained in the season’s quadrivalent inactivated influenza vaccine (QIV) and LAIV4, but not in the trivalent inactivated influenza vaccine (TIV), which contained only the B/Victoria lineage vaccine virus [3].

Several areas of concern in relation to vaccine effectiveness (VE) have emerged in recent seasons. Firstly, although the UK has found evidence of relatively good LAIV VE and continues to recommend its preferential use [4], in the United States (US) where there has been a longstanding paediatric influenza vaccination programme using both LAIV and inactivated influenza vaccine (IIV), reduced LAIV VE against influenza A(H1N1)pdm09 was reported by the US Centers for Disease Control and Prevention (CDC) [5]. This led to a recommendation from the Advisory Committee on Immunization Practice (ACIP) that LAIV should not be used in the US from 2016 until 2018. Questions were raised about what might explain these observations, such as reduced replicative ability of the A(H1N1)pdm09 strain [6]. Secondly, reductions in IIV VE particularly in older persons, a highly vaccinated population in the UK, have been previously noted, particularly against influenza A(H3N2), with several explanations proffered, including egg adaption of vaccine viruses, which may affect their antigenicity [7]. Finally, the majority of vaccinated adults at clinical risk were receiving TIV rather than QIV in the UK programme. Mismatches of the vaccine B-lineage virus compared with the predominant circulating influenza B virus lineage have been reported, which raises questions on the optimal vaccine to use in influenza vaccine programmes [4].

The 2017/18 influenza season in the UK was characterised by the co-circulation of both influenza A(H3N2) and influenza B, with some circulation of A(H1N1)pdm09. A large number of respiratory outbreaks in highly vaccinated populations were reported, particularly in long-term care facilities. In addition, increased admissions to hospital and excess mortality especially among older adult age groups were noted despite vaccine uptake levels of > 70% in ≥ 65 year olds [8]. The UK has a well-established system to monitor influenza VE each season based upon sentinel swabbing in primary care [9]. This paper presents the end-of-season 2017/18 VE findings for laboratory-confirmed infection in primary care across all age groups, with a focus on LAIV4 in children and IIV in adult age groups.

## Methods

### Design of the study

The test-negative case–control (TNCC) design was used to estimate VE, with the study undertaken in the registered population of five sentinel general practice surveillance networks across the UK, all of which undertake respiratory swabbing according to a standard protocol. Details of these schemes have been outlined previously [4]. The five sentinel schemes are: the Royal College of General Practitioners (RCGP) Research and Surveillance Centre (RSC) network, the Public Health England (PHE) Specialist Microbiology Network (SMN) and the national sentinel schemes of Northern Ireland, Scotland and Wales.

The study took place from 1 October 2017 – when respiratory swabbing was started – until 15 April 2018. The study population was patients presenting to their general practitioner (GP) during the study period with an acute influenza-like illness (ILI), who the GP obtained consent from and swabbed during the consultation. A case of ILI was defined as an individual who presented with an acute respiratory illness with physician-diagnosed fever or complaint of feverishness in the previous 7 days [9]. The combination of acute onset, cough and systemic symptoms (fever, headache, myalgia etc.) was recommended as a guide to diagnosis. Participating GPs were asked to invite persons presenting with ILI to provide a swab for diagnosis, with swabbing undertaken regardless of vaccination status. Cases were patients who tested positive for seasonal influenza A or B virus by real-time PCR. Controls were patients with the same symptoms who tested negative for influenza A or B virus.

During the consultation, the GP completed a standard questionnaire. This collected demographic (age and sex), clinical (date of onset and history of fever) and epidemiological information from patients including vaccination status. Vaccine history, including date of vaccination was obtained mainly from patient records. Vaccine type (LAIV4-intranasal; IIV injectable) was specified on the form. Additional information was collected for those ≥ 18 years of age on whether vaccination was with QIV or TIV. Persons in the study were categorised according to Department of Health defined risk categories for influenza vaccination [2]. High risk was determined by the presence of well recognised risk morbidities recorded in the electronic health record for the patient concerned [2]. In addition, it was noted whether the general practice was in a pilot area for England-based paediatric immunisation schemes, where all primary school age children were offered LAIV4 vaccine.

Patients were defined as vaccinated if they were reported to have received the 2017/18 seasonal vaccine at least 14 days before first onset of symptoms. Patients were excluded if they were vaccinated less than 14 days before symptom onset. If date of vaccination was unknown it was assumed to be 15/10/2017, which was the median of all known vaccination dates this season: the approach used in prior seasons [4].

Registered patients were excluded if they (or their parent/guardian) had expressed a wish to be; or the practice used one of the codes that indicate the patient may not want to share data (e.g. no consent for electronic record sharing). The opt out of sharing data was 2.25%.

### Detection and characterisation of influenza viruses in sentinel-surveillance- and non-sentinel samples

Combined throat and nose swabs taken from GPs are sent from the sentinel GP surveillance networks to their usual laboratory. Influenza laboratory confirmation was undertaken using comparable real-time PCR methods able to detect circulating influenza A and B viruses [10]. All laboratories sent influenza virus positive samples to the reference laboratories for further characterisation.

Isolation of Influenza viruses was attempted from all suitable PCR positive samples, from both sentinel GP practices and also non-sentinel schemes using Madin–Darby canine kidney epithelial (MDCK) cells or MDCK cells containing the cDNA of human 2,6-sialtransferase (SIAT1) cells [11,12].

Virus isolates with a haemagglutination titre ≥ 40 were characterised antigenically using post-infection ferret antisera in haemagglutination inhibition (HI) assays, with guinea pig (A(H3N2) viruses) or turkey (influenza B viruses) red blood cells [12]. Reference virus strains used for HI assays included 2017/18 vaccine strains [3] and other A(H3N2) and influenza B reference strains grown in embryonated chicken eggs and tissue culture cells.

Nucleotide sequencing of the haemagglutinin (HA) gene of a subset of influenza A(H3N2) and influenza B viruses selected to be representative of the range of the patients’ age, date of sample collection, geographical location and antigenic characterisation of the virus isolate, if performed, was undertaken. Phylogenetic trees of the HA gene of A(H3N2) and influenza B viruses were constructed with a neighbour-joining algorithm available in the Molecular Evolutionary Genetics Analysis (MEGA) 7 software (http://www.megasoftware.net) [13].

HA sequences from reference strains used in the phylogenetic analysis were obtained from the EpiFlu database of the Global Initiative on Sharing All Influenza Data (GISAID) (www.gisaid.org) (Supplement). The HA sequences generated for this study and used in the phylogenetic analysis, were deposited in GISAID under the following accession numbers: A(H3N2) viruses: EPI1112284, EPI1112292, EPI1112308, EPI1112348, EPI1112444, EPI1112484, EPI1112492, EPI1112500, EPI1112532, EPI1112596, EPI1112612, EPI1112620, EPI1112636, EPI1112652, EPI1112782, EPI1112788, EPI1139067, EPI1139075, EPI1139123, EPI1139155, EPI1139339, EPI1139347, EPI1139379, EPI1139490, EPI1139578, EPI1139646, EPI1139681, EPI1144477, EPI1144533, EPI1144549, EPI1144573, EPI1144589, EPI1144613, EPI1144661, EPI1144701, EPI1144709, EPI1144725, EPI1144845, EPI1144885, EPI1144925, EPI1144957, EPI1144965, EPI1144997, EPI1152027, EPI1152059, EPI1152227, EPI1152251, EPI1152275, EPI1152291, EPI1152451, EPI1152507, EPI1152623, EPI1152631, EPI1152687, EPI1152695, EPI1152711, EPI1152734, EPI1152736, EPI1152756, EPI1173388, EPI1173668, EPI1173700, EPI1173732, EPI1173756, EPI1173789, EPI1173979, EPI1173993, EPI1173999, EPI1174019; influenza B viruses: EPI1112540, EPI1112572, EPI1112580, EPI1112765, EPI1112790, EPI1112792, EPI1112798, EPI1139027, EPI1139163, EPI1139203, EPI1139299, EPI1139427, EPI1139482, EPI1139514, EPI1139538, EPI1139586, EPI1139666, EPI1144501, EPI1144509, EPI1144565, EPI1144605, EPI1144629, EPI1144637, EPI1144741, EPI1144797, EPI1144837, EPI1144909, EPI1144917, EPI1145005, EPI1152075, EPI1152091, EPI1152147, EPI1152155, EPI1152307, EPI1152403, EPI1152411, EPI1152483, EPI1152491, EPI1152523, EPI1152639, EPI1152679, EPI1152703, EPI1152745, EPI1152751, EPI1152760, EPI1173244, EPI1173284, EPI1173316, EPI1173340, EPI1173348, EPI1173356, EPI1173372, EPI1173420, EPI1173428, EPI1173444, EPI1173468, EPI1173476, EPI1173484, EPI1173516, EPI1173532, EPI1173556, EPI1173580, EPI1173676, EPI1173983, EPI1173997, EPI1199126, EPI811586.

### Seroepidemiological survey

Annual sero-surveys were carried out in England using residual sera collected and submitted to the PHE Sero-epidemiology Unit (SEU) during the 2016 and 2017 summers. The PHE SEU archive is an opportunistic collection of residual serum samples from routine microbiological testing, submitted voluntarily each year from laboratories throughout England, with samples anonymised and permanently unlinked from any patient identifying information with only age, sex, date of collection and contributing laboratory retained. A total of 780 residual sera from the SEU were collected during the 2017 summer period (after the previous and before start of the current influenza season) and 1,000 samples in summer 2016. Samples were randomly selected with constraints to provide an even distribution by age and region. The sample size was chosen to enable reasonable precision of estimates (95% confidence interval (CI) width less than +/− 10%) within each age group.

Laboratory analysis was focused on detection of antibody to influenza A(H3N2) and B/Yamagata viruses. Serum samples were analysed for presence of A(H3N2) antibody to representative seasonal influenza strains as indicated by the vaccine composition for 2017/18 using guinea pig erythrocytes for A(H3N2) and turkey erythrocytes for influenza B as previously described. Antigen was grown in eggs and tissue culture (MDCK cells) to examine the issue of potential egg adaptation and the influenza B antigen diethyl-ether extracted.

Sera were analysed in single titrations in 96-well format by performing doubling serum dilutions starting at 1:10 up to 1:1,280, with virus input adjusted to 4 haemagglutination forming units (4 HAU).

### Statistical methods

The swabbing results were analysed using a test-negative design for VE. The odds ratio (OR) of being vaccinated between cases and controls was used to calculate the crude VE as (1 − OR) x 100%. We performed a multivariable logistic regression, as previously [4], to adjust VE for potential confounders with influenza laboratory results as the outcome and influenza vaccination status as the linear predictor. Estimates were calculated adjusting for age (by < 2, 2–11, 12–17, 18–44, 45–64 and ≥ 65 years age groups), month of onset of symptoms, surveillance scheme, risk-group, sex, and residence in an area where a primary school programme was in place. Stratification was by age 2–17, 18–64 and ≥ 65 years and was split by vaccine type: LAIV4/QIV within those aged 2–17 years and QIV/TIV for those aged ≥ 18 years. The effect of prior season vaccination was also described by calculating all the VEs (vaccinated both 2017/18 and 2016/17, only 2016/17 and only 2017/18) and comparing to not vaccinated in either season. Decline in VE was assessed by stratification in the model by time since vaccination (vaccinated within 3 months of onset and ≥ 3 months before onset), and also by stratification by month of onset (October–December/January–April). To minimise inclusion of underpowered results, VE estimates where the upper and lower 95% CI respectively extended below −50% and above 80% were excluded.

Sensitivity analyses were undertaken – specifically including all swabs no matter how long after onset they had been taken; then a model including those vaccinated within 14 days as unvaccinated.

For statistical analysis of the seroprevalence data, serum titres below the detection limit (< 10) were assigned a numeric value of five, while sera above the previously determined threshold titre ≥ 40 were considered seropositive for the purpose of analysis and all available titres were transformed into log_2_titres. Proportions with positive titres with 95% CI by age group are presented. To compare A(H3N2) proportions positive for antigen grown in tissue culture compared with egg, McNemar’s chi-squared test for paired data was used on those samples tested by both assays. 

### Ethical statement

The collection of sera in the PHE SEU archive has undergone ethical review (REC reference: 05/Q0505/45). The collection and analysis of swab forms according to positivity was undertaken as part of routine influenza surveillance, with swab test-results relayed back to sentinel GPs to assist in clinical management. The collection of the clinical data accords with routine usual practice in public health. Specific ethical approval was not necessary.

## Results

### Characteristics of study patients

**Figure 1:**
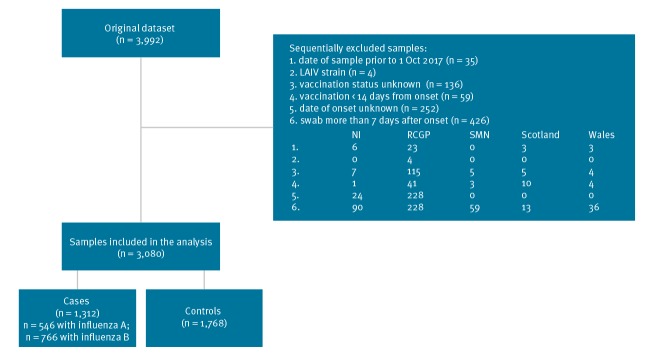
Swabbing results of patients with influenza-like illness in primary care in the United Kingdom, October 2017–April 2018 (n = 3,992 patients swabbed)

LAIV4: quadrivalent live attenuated influenza vaccine; NI: Northern Ireland national sentinel scheme; RCGP: Royal College of General Practitioners’ Research and Surveillance Centre; SMN: Public Health England Specialist Microbiology Network.

During the study, 3,992 persons were sampled in the participating sentinel primary care practices and were tested. A total of 912 samples were excluded: the reasons for study exclusion are summarised in Figure 1, with swabbing more than 7 days after onset the main explanation. Four samples were excluded as LAIV vaccine virus was detected. The details of the 3,080 samples remaining stratified according to the swab result and by vaccination are described in Tables 1 and 2. A total of 149 samples had date of vaccination imputed. There were 1,768 controls and 1,312 cases, of whom 546 were influenza A (431 H3N2, 22 A unknown and 96 (H1N1)pdm09, and 766 were influenza B, with a small number of multiple infections (two cases).

**Table 1:** Characteristics of influenza A and B cases and controls, United Kingdom, October 2017–April 2018 (n = 3,080)

Characteristics	Control (n = 1,768)	Cases by influenza type (and subtype if known) (n = 1,315)^a^				Total	p value
		B (n = 766)	A(H1N1) (n = 96)	A(H3N2) (n = 431)	A (unknown) (n = 22)		
**Age in years**							
0–1	153	7	7	8	0	175	< 0.0001
2–11	212	71	22	58	0	363	
12–17	98	66	5	28	4	201	
18–44	587	267	31	143	7	1,035	
45–64	447	261	22	122	10	862	
≥ 65	270	94	9	72	1	446	
Missing	1	0	0	0	0	1	
**Sex**							
Female	1,083	416	49	247	14	1,809	0.004
Male	671	346	47	184	8	1,256	
Missing	14	4	0	0	0	18	
**Database**							
Northern Ireland	99	85	6	66	8	264	< 0.0001
RCGP	1,203	484	76	189	0	1,952	
SMN	86	51	6	16	4	163	
Scotland	266	79	5	135	10	495	
Wales	114	67	3	25	0	209	
**Risk group**							
No	1,075	553	62	258	11	1,959	< 0.0001
Yes	545	139	25	137	7	853	
Missing	148	74	9	36	4	271	
**Onset to swab in days**							
0–1	240	78	13	85	5	421	< 0.0001
2–4	867	458	59	252	10	1,646	
5–7	661	230	24	94	7	1,016	
**Vaccination status**							
Unvaccinated	1,273	594	78	280	18	2,243	< 0.0001
Vaccinated (14–91 days ago)	297	75	10	78	2	462	
Vaccinated (> 91 days ago)	198	97	8	73	2	378	
**Month of event**							
October	270	2	0	4	2	278	< 0.0001
November	310	27	4	18	2	361	
December	344	169	19	125	4	661	
January	509	374	39	167	10	1,099	
February	206	147	24	67	3	447	
March	110	45	8	48	1	212	
April	19	2	2	2	0	25	
**Pilot area (SMN and RCGP RSC only)**							
No	1,391	599	84	226	3	2,303	< 0.0001
Yes	377	167	12	205	19	780	
**Vaccination status (for 2–17 year olds only)**							
Unvaccinated	252	127	26	62	4	471	0.52
Injection (QIV)	9	1	0	1	0	11	
Intranasal/LAIV4	49	9	1	23	0	82	

LAIV4: quadrivalent live attenuated influenza vaccine; QIV: quadrivalent inactivated influenza vaccine; RCGP RSC: Royal College of General Practitioners’ Research and Surveillance Centre scheme; SMN: Public Health England Specialist Microbiology Network.

^a^ While the study comprised 1,312 cases of influenza, 1,315 are presented in the table, because there were two cases of infection with multiple types or subtypes. One of these cases was infected by influenza A(H1N1)pdm09 and, in addition A(H3N2) and B (thus accounting, for two additional cases in the total number of cases by type/subtype) and the other was infected by A(H1N1)pdm09 and also A(H3N2) (thus accounting for one additional case in the total number of cases by type/subtype).

A total of 149 samples had date of vaccination imputed.

**Table 2:** Details of vaccination status for key demographic and clinical variables, United Kingdom, October 2017–April 2018 (n = 3,080)

Characteristics	Not vaccinated (n = 2,242)	Vaccinated (n = 838)	Total	p value
**Age in years**				
< 2	172	3	175	< 0.0001
2–11	270	93	363	
12–17	187	14	201	
18–44	886	148	1,034	
45–64	619	243	862	
≥ 65	108	336	444	
Missing	0	1	1	
**Sex**				
Female	1,279	527	1,806	0.004
Male	948	308	1,256	
Missing	15	3	18	
**Surveillance scheme**				
Northern Ireland	206	58	264	0.002
RCGP RSC	1,380	571	1,951	
SMN	116	47	163	
Scotland	371	122	493	
Wales	169	40	209	
**Risk group**				
No	1,665	294	1,959	< 0.0001
Yes	376	474	850	
Missing	201	70	271	
**Onset to swab in days**				
0–1	315	106	421	0.49
2–4	1,185	458	1,643	
5–7	742	274	1,016	
**Pilot area (RCGP RSC and SMN only)**				
No	1,650	652	2,302	0.014
Yes	592	186	778	
**Month of event**				
October	244	34	278	< 0.0001
November	290	69	359	
December	478	183	661	
January	773	326	1,099	
February	309	138	447	
March	136	75	211	
April	12	13	25	
**Cases infected with strains characterised by sequencing/phylogenetic analysis**				
Influenza A				
H3N2 - 3C.2a3 - subgroup 2	1	1	2	0.02
H3N2 - 3C.2a2 - subgroup 3	49	64	113	
H3N2 - 3C.2a - subgroup NA	1	0	1	
H3N2 - 3C.2a1a - subgroup 4	3	0	3	
H3N2 - 3C.2a1b - subgroup 5	18	7	25	
H3N2 - 3C.2a1 - subgroup NA	8	3	11	
H3N2 - 3C.3a	4	3	7	
H1N1 – 6B.1 clade	35	12	47	N/A
Influenza B				
B/Yamagata	300	106	406	0.26
B/Victoria	0	1	1	

RCGP RSC: Royal College of General Practitioners’ Research and Surveillance Centre scheme; SMN: Public Health England Specialist Microbiology Network.

### Influenza strains detected during the 2017/18 season

**Figure 2:**
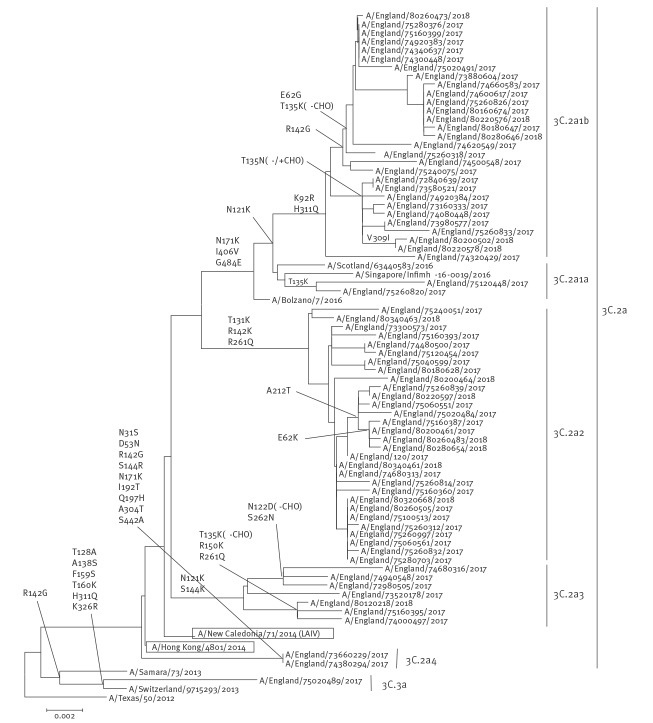
Phylogenetic analysis of the haemagglutinin sequences of influenza A(H3N2) viruses detected in the United Kingdom, July 2017–April 2018^a^

LAIV: live attenuated influenza vaccine ; QIV: quadrivalent inactivated influenza vaccine; TIV: trivalent inactivated influenza vaccine.

^a^ The sequences are obtained from all sources (i.e. sentinel surveillance and non-sentinel schemes) so some of the sequences are from as early as July 2017.

Figure 2 shows the phylogenetic analysis of the HA sequences for A(H3N2) 2017/18 viruses. Genetic characterisation of 778 A(H3N2) influenza viruses from all sources (i.e. sentinel surveillance and non-sentinel schemes) since week 40 showed that the majority (747; 96%) belong to HA genetic subclade 3C.2a, with 199 (27%) of these 747 viruses belonging to a cluster within this genetic subclade designated as 3C.2a1, and the others belonging to other clusters in 3C.2a, designated as 3C.2a2, 3C.2a3 and 3C.2a4. The remaining 31 A(H3N2) viruses (4%) fell in HA subclade 3C.3a. The northern hemisphere 2017/18 influenza A(H3N2) vaccine strain A/HongKong/4801/2014 belonged in genetic subclade 3C.2a and its relatedness to the circulating strains is shown in Figure 2.

The H3 haemagglutinin sequences obtained in England in 2017 and 2018 are depicted on the tree. The vaccine strains (A/HongKong/4801/2014 for TIV and QIV; A/NewCaledonia/71/2014 for LAIV) are boxed; reference viruses are represented in italics. Amino acid substitutions are depicted at the nodes. ‘-/+CHO’ indicates the addition or deletion of a potential glycosylation site. The names of the subclades and clusters to which the sequences belong to are indicated on the right hand side of the tree. The scale is proportional to number of nucleotide substitutions per site.

**Figure 3:**
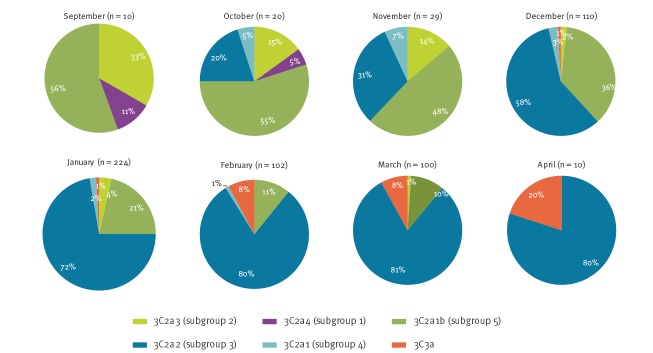
Frequency of H3N2 haemagglutinin genetic groups by month, England, September 2017–April 2018 (n = 605)

The emergence of subgroups and temporal differences in the distribution of viruses within both 3C.2a and 3C.2a1 has been observed over the season in viruses from all sources (Figure 3). Early in the season during October and November, viruses in 3C.2a1, belonging mainly in subgroup 3C.2a1b (clade 5) accounted for 55–60% of the A(H3N2) viruses from all sources characterised genetically. During December 2017 to April 2018, the proportion of viruses belonging to the subclade 3C.2a2 (subgroup 3) become the dominant circulating A(H3N2) subgroup by the end of the season. This season’s A(H3N2) viruses were again difficult to type by HI analysis with ferret antisera, and only 24 influenza A(H3N2) viruses from all sources were antigenically characterised since week 40 2017, representing a minority of the detections and thus a potential bias in the available antigenic data. The viruses antigenically analysed showed better reactivity to ferret antiserum raised to tissue culture grown A/HongKong/4801/2014 virus, than with antiserum derived from egg propagated A/HongKong/4801/2014 virus. All 24 antigenically characterised viruses were also genetically characterised, with 15 belonging in genetic group 3C.2a, including 10 within 3C.2a2 (subgroup3) and five in 3C.2a1 (subgroup 4) and nine H3N2 isolates belonging in subclade 3C.3a.

Genetic groups definition as suggested by NextFlu/Crick Feb 2018 (in brackets, previous definition suggested by NextFlu/ECDC Sept 2017).

**Figure 4:**
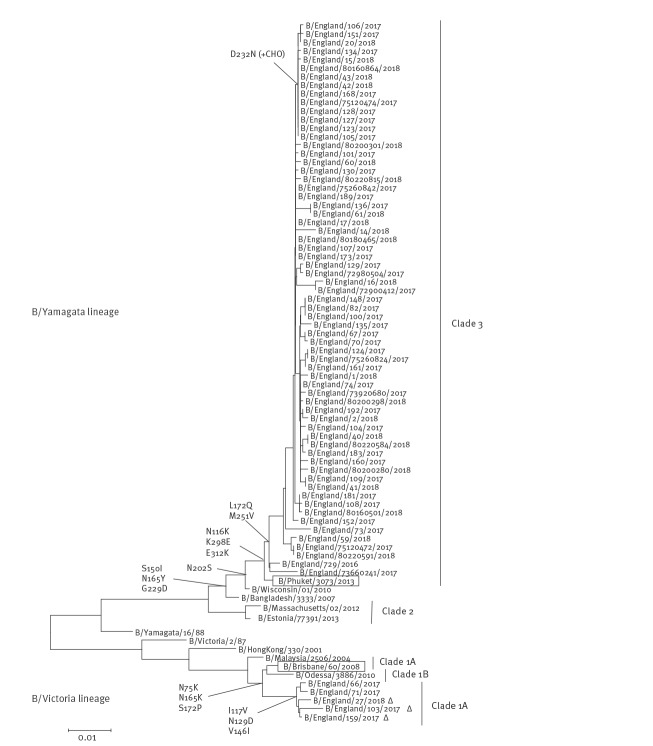
Phylogenetic analysis of the haemagglutinin sequences of influenza B viruses detected in the United Kingdom, July 2017–April 2018

LAIV: live attenuated influenza vaccine; QIV: quadrivalent inactivated influenza vaccine; TIV: trivalent inactivated influenza vaccine.

^a^ The sequences are obtained from all sources (i.e. sentinel surveillance and non-sentinel schemes) so some of the sequences are from as early as July 2017.

Genetic characterisation of 688 influenza B viruses from all sources was completed. A total of 682 (99%) viruses were classified as belonging to the B/Yamagata/16/88-lineage, genetically similar to B/Phuket/3073/2013, the influenza B/Yamagata/16/88-lineage component of 2017/18 northern hemisphere quadrivalent vaccine [14]. Six (1%) were classified as falling in the B/Victoria/2/87-lineage; genetically similar to B/Brisbane/60/2008 (the influenza B/Victoria/2/87-lineage component of 2017/18 northern hemisphere trivalent and quadrivalent vaccines [14]) falling within genetic clade 1A, but with five of these belonging within a subgroup in clade 1A characterised by deletion of two amino acids in the HA. The relationship of the HA genes of the circulating strains analysed compared with the vaccine strains is shown in Figure 4. A total of 489 influenza B viruses were isolated and antigenically characterised since week 40 2017; 485 (99%) viruses were characterised as belonging to the B/Yamagata/16/88-lineage and antigenically similar to B/Phuket/3073/2013. Of the viruses characterised as belonging to the B/Victoria/2/87-lineage, one virus was antigenically similar to B/Brisbane/60/2008, with the double deletion subgroup viruses characterised as antigenically distinct from B/Brisbane/60/2008.

The influenza B haemagglutinin sequences obtained in England in 2017 and 2018 are depicted on the tree. The vaccine strains (B/Brisbane/60/2008 for TIV, QIV and LAIV; B/Phuket/3073/2013 for QIV and LAIV) are boxed; reference viruses are represented in italics. Amino acid substitutions are depicted at the nodes. ‘+CHO’ indicates the addition of a potential glycosylation site. The names of the clades to which the sequences belong to are indicated on the right hand side of the tree. The scale is proportional to number of nucleotide substitutions per site.

### Model fitting for vaccine effectiveness estimation

When estimating VE, age group, sex, time period (defined by month of sample collection), surveillance scheme, risk group and primary school age pilot programme area were adjusted for in a multivariable logistic regression model. All variables that were adjusted for were significantly associated with a positive swab (Table 1). The number and proportion vaccinated for these variables are shown in Table 2. Only age, month of onset and risk-factor were confounders for the vaccine effects (changing the overall estimate by more than 5%).

The crude and adjusted VE estimates against all influenza, influenza A(H3N2), influenza A(H1N1)pdm09 and B are shown in Table 3. For any influenza (A or B), the crude VE for all ages was 9.0%; the adjusted VE point estimate of influenza vaccine against any laboratory-confirmed infection was 15.0% (95% CI: −6.3 to 32.0). Further sensitivity analyses were undertaken. Firstly, including all swabs no matter how long after onset they had been taken made less than 3% difference to the overall VE point estimate. Then a model including those vaccinated within 14 days as unvaccinated and including all swabs regardless of time since onset of symptoms found again < 3% difference to the VE point estimate.

**Table 3:** Vaccine effectiveness estimates for influenza by subtype, clade, age group and vaccine type, United Kingdom, October 2017–April 2018 (n = 3,080)

Characteristics	Cases		Controls		Crude VE (95% CI)	Adjusted^c^ VE (95% CI)
	Vaccinated^a^	Unvaccinated	Vaccinated^b^	Unvaccinated		
**Influenza A and B, by age group in years and vaccine type for 2–17 year olds**						
All age	343	969	495	1,273	9.0 (−6.9 to 22.5)	15.0 (−6.3 to 32.0)
2–17 (QIV)	2	213	9	244	NR	NR
2–17 (LAIV4)	33	213	49	244	22.9 (−24.4 to 52.2)	26.9 (−32.6 to 59.7)
18–64	166	696	225	809	14.2 (−7.4 to 31.5)	12.2 (−16.8 to 34.0)
≥ 65	136	38	201	70	−24.6 (−95.7 to 20.6)	10.1 (−54.8 to 47.8)
**Influenza A by age group in years and vaccine type for 2–17 year olds**						
All age	172	375	495	1,273	−18.0 (−45.3 to 4.2)	4.5 (−27.4 to 28.5)
2–17 (QIV)	1	86	9	244	NR	NR
2–17 (LAIV4)	24	86	49	244	−39.0 (−140.1 to 19.6)	−1.8 (−108.1 to 50.2)
18–64	76	258	225	809	−5.9 (−42.3 to 21.2)	4.4 (−39.9 to 34.6)
≥ 65	65	16	201	70	−41.5 (−160.6 to 23.2)	10.3 (−82.1 to 55.8)
**Influenza A(H3N2) by age group in years and vaccine type for 2–17 year olds**						
All age	151	280	495	1,273	−38.7 (−73.4 to −10.9)	−16.4 (−59.3 to 14.9)
2–17 (QIV)	1	59	9	244	NR	NR
2–17 (LAIV4)	23	59	49	244	−94.1 (−243.7 to −9.6)	−75.5 (−289.6 to 21)
18–64	67	198	225	809	−21.7 (−66.5 to 11.1)	−14.7 (−72.7 to 23.8)
≥ 65	57	15	201	70	−32.3 (−148.6 to 29.6)	16.8 (−74.2 to 60.3)
**Influenza A(H1N1)pdm09 by age group in years and vaccine type for 2–17 year olds**						
All age	18	78	495	1,273	40.7 (−0.1 to 64.8)	66.3 (33.4 to 82.9)
2–17 (QIV)	0	23	9	244	NR	NR
2–17 (LAIV4)	1	23	49	244	78.3 (−64.1 to 97.1)	90.3 (16.4 to 98.9)
18–64	6	47	225	809	54.1 (−8.7 to 80.6)	69.1 (11.4 to 89.2)
≥ 65	8	1	201	70	NR	NR
**Influenza B by age group in years and type of vacci**n**e**						
All age	172	594	495	1,273	25.5 (9.1 to 39.0)	24.7 (1.1 to 42.7)
2–17 (QIV)	1	127	9	244	NR	NR
2–17 (LAIV4)	9	127	49	244	64.7 (25.9 to 83.2)	60.8 (8.2 to 83.3)
18–64	90	438	225	809	26.1 (3.2 to 43.6)	18.2 (−15.1 to 41.9)
≥ 65	72	22	201	70	−14.0 (−97.5 to 34.2)	13.2 (−68.4 to 55.2)
TIV (≥ 18)	49	460	92	879	−1.8 (−46.5 to 29.3)	1.9 (−63.6 to 41.2)
QIV (≥ 18)	1	460	6	879	NR	NR
**By influenza virus clade/lineage (all age)**						
B/Yamagata	106	300	495	1,273	9.1 (−16.0 to 28.8)	16.9 (−17.0 to 41.0)
H1/6b1	12	35	495	1,273	11.8 (−71.2 to 54.6)	57.9 (−2.0 to 82.6)
H3/3C2a2 (subgroup 3)	64	49	495	1,273	−235.9 (−394.3 to −128.3)	−95.2 (−230.6 to −15.3)
H3/3C2a1b (subgroup 5)	7	18	495	1,273	−0.01 (−140.9 to 58.5)	29.1 (−116 to 76.7)
H3/3C3a	3	4	495	1,273	NR	NR

CI: confidence interval; LAIV4: quadrivalent live attenuated influenza vaccine; NR: not reported; QIV: quadrivalent inactivated vaccine; TIV: trivalent inactivated vaccine; VE: vaccine effectiveness.

^a^ Six vaccinated cases aged between 2–17 years (three infected with influenza A(H3N2) and three infected with A(H1N1)pdm09) had no information on the type of vaccine (QIV or TIV) they received, so they are not included in the analyses presented in the table.

^b^ Eight vaccinated controls aged between 2–17 years had no information on the type of vaccine (QIV or TIV) they received, so they are not included in the analyses presented in the table.

**^c^** Adjusted for age group, risk-group, sex, month, pilot area and surveillance scheme.

### Influenza A(H3N2)

The all-age adjusted VE was −16.4% (95% CI: −59.3 to 14.9) for A(H3N2) (Table 3). Examining clade- and lineage-specific estimates demonstrated overlapping CIs (Table 3).

#### Vaccine effectiveness in adults

Table 3 shows the adjusted VE against influenza A(H3N2) for inactivated vaccine (IIV) in 18–64 year olds (−14.7%; 95% CI: −72.7 to 23.8) and ≥ 65 year olds (16.8%; 95% CI: −74.2 to 60.3) with no evidence of significant effectiveness in either group. In relation to vaccination in the prior season in those ≥ 18 years of age (Table 4), the VE point estimate were low in all strata and differences were non-significant (p value = 0.69).

**Table 4:** Adjusted vaccine effectiveness estimates for influenza A(H3N2) by prior vaccination status and subtype in children 2–17 years of age (LAIV only) and adults ≥ 18 years, United Kingdom, October 2017–April 2018 (n = 1,888)

Vaccination status	Cases	Controls	Adjusted^a^ VE (95% CI)
**2–17 year olds (LAIV only)**			
Unvaccinated in 2016/17 and 2017/18	49	196	Reference
Vaccinated only in 2016/17	5	33	NR
Vaccinated only in 2017/18	10	20	−139.9 (−615.1 to 19.5)
Vaccinated in 2016/17 and 2017/18	11	20	−61.8 (−372 to 44.5)
≥ **18 year olds**			
Unvaccinated in 2016/17 and 2017/18	189	743	Reference
Vaccinated only in 2016/17	16	96	12.9 (−68.3 to 55.0)
Vaccinated only in 2017/18	20	76	−2.6 (−85.8 to 43.4)
Vaccinated in 2016/17 and 2017/18	94	310	−9.3 (−64.8 to 27.5)

CI: confidence interval; LAIV: live attenuated influenza vaccine; NR: not reported; VE: vaccine effectiveness.

^a^ Adjusted for age group, risk-group, sex, month, pilot area and surveillance scheme.

#### Vaccine effectiveness in children

The crude and adjusted VE against A(H3N2) in children 2–17 years of age for LAIV4 is shown in Table 3 with no evidence of significant effectiveness. There were sparse data to undertake analysis of VE for QIV in children. Table 4 shows the influence of LAIV4 vaccination in 2–17 year olds in the prior season, the differences were non-significant (p value = 0.17).

### Influenza A(H1N1)pdm09

The adjusted VE was 66.3% (95% CI: 33.4 to 82.9) for A(H1N1)pdm09 for all ages (Table 3).

#### Vaccine effectiveness in adults

Table 3 shows an adjusted VE against A(H1N1)pdm09 for inactivated vaccine (IIV) in 18–64 year olds of 69.1% (95% CI: 11.4 to 89.2).

#### Vaccine effectiveness in children

The adjusted VE against A(H1N1)pdm09 in 2–17 year olds for LAIV4 (Table 3) was 90.3% (95% CI: 16.4 to 98.9). There were sparse data to undertake analysis of VE for QIV in children or according to prior season vaccine history in adults and children.

### Influenza B

The overall all-age adjusted VE estimate against influenza B was 24.7% (95% CI: 1.1 to 42.7) (Table 3), compared with 16.9% (95% CI: −17.0 to 41.0) against only B/Yamagata/16/88-lineage (Table 3).

#### Vaccine effectiveness in adults

The adjusted VE against influenza B for 18–64 years olds for IIV (Table 3) was 18.2% (95% CI: −15.1 to 41.9) and was 13.2% (95% CI: −68.4 to 55.2) for those ≥ 65 years of age. Statistically significant protection was not seen in either age group. There were sparse data to undertake analysis of VE for QIV in adults, though effectiveness against TIV only was low at 1.9% (95% CI: −63.6 to 41.2) (Table 3). In relation to vaccination in the prior season in those ≥ 18 years of age (Table 5), the adjusted VE estimates were similar regardless of prior vaccine history.

**Table 5:** Adjusted vaccine effectiveness estimates for influenza B by prior vaccine status and subtype in children 2–17 years of age (LAIV4 only) and adults ≥ 18 years, United Kingdom, October 2017–April 2018 (n = 2,184)

Vaccination status	Cases	Controls	Adjusted^a^ VE (95% CI)
**2–17 year olds (LAIV only)**			
Unvaccinated in 2016/17 and 2017/18	115	196	Reference
Vaccinated only in 2016/17	2	33	72.5 (−33.8 to 94.3)
Vaccinated only in 2017/18	4	20	NR
Vaccinated in 2016/17 and 2017/18	2	20	81.6 (10.9 to 96.2)
≥ **18 year olds**			
Unvaccinated in 2016/17 and 2017/18	411	743	Reference
Vaccinated only in 2016/17	15	96	40.0 (−13.0 to 68.1)
Vaccinated only in 2017/18	24	76	35.3 (−10.8 to 62.3)
Vaccinated in 2016/17 and 2017/18	117	310	12.1 (−24.6 to 38.0)

CI: confidence interval; LAIV: live attenuated influenza vaccine; NR: not reported; VE: vaccine effectiveness.

^a^ Adjusted for age group, risk-group, sex, month, pilot area and surveillance scheme.

#### Vaccine effectiveness in children

The adjusted VE against influenza B in children 2–17 years of age for LAIV4 was 60.8% (95% CI: 8.2 to 83.3) (Table 3). Table 5 shows the influence of prior season LAIV4 vaccination in 2–17 year olds on influenza B. The VE point estimate was high and similar regardless of prior season vaccine history.

### Vaccine effectiveness by time since vaccination and period

Table 6 shows the adjusted VE by time since vaccination and period. No significant difference was observed for influenza B, A(H3N2) or A(H1N1)pdm09.

**Table 6:** Adjusted vaccine effectiveness estimates for influenza by influenza subtype and time since vaccination and period, United Kingdom, October 2017–April 2018 (n = 3,080)

Time elapsed since vaccination at symptom onset or period of symptom onset	Cases		Controls		Adjusted^a^ VE (95% CI)
	Vaccinated	Unvaccinated	Vaccinated	Unvaccinated	
**Influenza A and B**					
3 months	163	969	297	1,273	18.7 (−7.7 to 38.6)
≥ 3 months	180	969	198	1,273	10.4 (−19.9 to 33.0)
Oct to Dec	83	291	203	721	−7.3 (−65.4 to 30.4)
Jan to Apr	260	678	292	552	20.3 (−3.9 to 38.9)
**Influenza A**					
3 months	89	375	297	1,273	−1.3 (−44.7 to 29.1)
≥ 3 months	83	375	198	1,273	9.5 (−32.4 to 38.1)
Oct to Dec	48	129	203	721	−43.4 (−157.0 to 20.0)
Jan to Apr	124	246	292	552	14.9 (−19.8 to 39.5)
**Influenza A(H3N2)**					
3 months	78	280	297	1,273	−17.6 (−73.7 to 20.4)
≥ 3 months	73	280	198	1,273	−15.4 (−73.4 to 23.2)
Oct to Dec	44	103	203	721	−71.0 (−227.4 to 10.7)
Jan to Apr	107	177	292	552	−3.6 (−49.7 to 28.3)
**Influenza H1N1pdm09**					
3 months	10	78	297	1,273	47.9 (−15.1 to 76.5)
≥ 3 months	8	78	198	1,273	78.8 (40.2 to 92.5)
Oct to Dec	4	19	203	721	NR
Jan to Apr	14	59	292	552	71.7 (36.8 to 87.4)
**Influenza B**					
3 months	75	594	297	1,273	33.5 (5.2 to 53.4)
≥ 3 months	97	594	198	1,273	16.1 (−18.8 to 40.8)
Oct to Dec	36	162	203	721	19.3 (−40.3 to 53.6)
Jan to Apr	136	432	292	552	25.9 (−1.8 to 46.0)

CI: confidence interval; NR: not reported; VE: vaccine effectiveness

**^a^** Adjusted for age group, risk-group,  sex, month, pilot area and surveillance scheme.

### Detection of antibody in population serum samples

**Figure 5:**
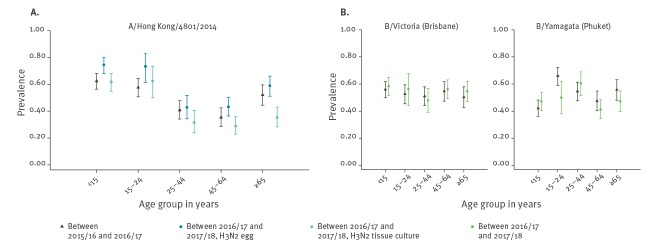
Antibody seroprevalence levels by age group against (A) A/Hong Kong/4801/2014 virus either grown in tissue culture or propagated in egg or (B) B/Yamagata and B/Victoria viruses, England, United Kingdom, 2016 and 2017 (n = 1,741)

Antibody prevalence levels to A/Hong Kong/4801/2014 virus were highest in the youngest (< 15 years and 15–24 year-old) age groups for egg-grown virus for samples taken in summer 2017. Although egg-grown A(H3N2) (‘vaccine-like’ virus) antibody levels were lower for 25–64 year olds, they were relatively higher in those aged ≥ 65 years old (Figure 5A) – which is the most highly vaccinated population. The pattern was similar for samples taken in summer 2016. Overall the 2017 seroprevalence was lower for A(H3N2) viruses grown in tissue culture compared with egg-grown across all age groups, with the mean differences by age group statistically significant in all ages except 15–24 year olds (Figure 5A). The difference in seropositivity proportions between the two laboratory assays (tissue culture minus egg-grown) and their corresponding CIs and p values for the same age groups are given as: < 15 years olds: −13% (95% CI: −19% to −7%, p < 0.001); 15–24 years old: −11% (−23% to 1%, p = 0.09); 25–44 years old: −11% (−20% to −2%, p = 0.02); 45–64 years old: −15% (−22% to −7%, p < 0.001); ≥ 65 years old: −24% (−32% to −15%, p < 0.001). 

Antibody prevalence levels to B/Yamagata were highest in young adults (15–24 years) and the elderly (≥ 65 years) in summer 2016 (Figure 5B). For the 2017 samples, antibody levels were lower for 15–24 and ≥ 65 year olds, but higher for < 15 and 25–44 year olds (Figure 5B). The age-specific pattern for B/Victoria was similar between the two periods.

## Discussion

This study finds overall a low influenza VE during a season that saw co-circulation of influenza B with a B-lineage mismatch comparing to trivalent vaccine and influenza A(H3N2) with a new dominant genetic subgroup. We demonstrate significant effectiveness against influenza B in children who received the quadrivalent influenza vaccine, but poor effectiveness against influenza B in adults, particularly those who received trivalent vaccine. No significant VE against A(H3N2) was seen in either adults or children regardless of whether vaccine was live attenuated or inactivated. A number of variants of A(H3N2) circulated during the course of the winter, with subgroups present at the start of the season gradually being replaced by different subgroups later. There was evidence of reduced population immunity to tissue-culture-adapted A(H3N2) vaccine virus, which is more representative of the World Health Organization (WHO) recommended vaccine virus compared to egg-propagated virus [15]. Finally, we found good protection against influenza A(H1N1)pdm09 in both adults and children, especially in those who had received live attenuated influenza vaccine.

There are several potential strengths to this study. The test-negative case–control design which is used, is a well-established approach to measure influenza VE in the UK as in many other countries. We used our standard method to provide comparability to previous season’s UK VE estimates. In addition, we triangulated VE and population seroprevalence data to provide important insights into the underlying explanations for this season’s observations. There are some limitations to the study; in particular only small numbers of children, contraindicated LAIV, had received QIV with consequent inability to provide reliable VE estimates for this group. Due to limited circulation, only limited VE estimates for A(H1N1)pdm09 could be confidently undertaken. Date of vaccination had to be imputed for some records. This is unlikely to have led to a large amount of misclassification as influenza circulation started after the majority of influenza vaccination had been completed by the end of November. The only patients that misclassification has a non-negligible chance of occurring for are those with onset in October (n = 9) or November (n = 11) with missing vaccination date. This is a very small proportion of the total numbers vaccinated.

The end-of-season VE estimation against all laboratory-confirmed influenza illness presenting in primary care found poor effectiveness, also in adults. The results, which are driven by the dominance of A(H3N2) and B are consistent with the mid- and end-of-season 2017/18 VE estimates published elsewhere in Europe and North America, many of whom experienced seasons with circulation of A(H3N2) [16-18]. Our findings of reduced VE against influenza B are discordant with a number of other settings in Europe and North America [16-18]. This observation of reduced VE is likely to be due to several factors.

Notably the 2017/18 season in the UK saw early circulation of influenza B with a lineage mismatch to the 2017/18 trivalent vaccine, where influenza B cases observed in the UK were mainly due to viruses belonging to the B/Yamagata lineage, with only a small proportion of B/Victoria lineage viruses detected [8]. There was evidence of good protection against influenza B in children who received the LAIV4 vaccine. However, poor effectiveness against influenza B was observed in adults, particularly when restricted to those vaccinated persons who had received the 2017/18 TIV. Unfortunately, there were sparse data to calculate a reliable VE estimate for QIV only. Our finding of poor TIV effectiveness against influenza B in adults is consistent with the epidemiology seen in the UK in 2017/18, with large numbers of influenza B cases hospitalised in highly vaccinated populations such as ≥ 65 year olds [8]. Our results were inconsistent with some studies elsewhere in Europe and Canada that did suggest a degree of cross-protection even though there was circulation of a lineage mismatch influenza B virus. These latter observations may be due to prior infection or vaccination with a B/Yamagata lineage virus in earlier seasons [19]. Although we were unable to estimate QIV effectiveness in adults, some published data do demonstrate superior effectiveness and cost-effectiveness of QIV compared with TIV [20,21]. More recent modelling by PHE based on this work has been conducted to understand the potential incremental benefit of QIV in adults in the presence of the UK childhood LAIV4 programme [22]. This work found that, once the programme in children of primary school age is fully established, there is still benefit from preferentially using QIV in at risk adults < 65 years of age, including pregnant women.

We found no evidence of significant effectiveness against influenza A(H3N2) with either inactivated or live attenuated influenza vaccine, all of which are manufactured on eggs. As the season progressed the majority of A(H3N2) viruses that were genetically characterised shifted from genetic subclade 3C.2a1b (subgroup 5) to subclade 3C.2a2 (subgroup 3) which comprised the majority of A(H3N2) vaccine failures observed this season. This compares to the 2016/17 UK influenza season that was also dominated by A(H3N2) though mainly of the 3C.2a1 subclade, where although effectiveness in the elderly was poor, there was still evidence by the end of the season of significant effectiveness of LAIV4 against A(H3N2) in children and of moderate protection in young adults [23]. Such evidence of poorer VE against A(H3N2), has been well recognised in recent seasons [24,25]. This is a complex and multifactorial problem. The A(H3N2) vaccine virus component has not changed between 2016/17 and 2017/18, and our results suggest that the divergence in results between the two seasons may be due to several factors including changes in the dominant circulating genetic subgroup. It is not possible to conclude if these genetic changes were significant antigenically. The analyses used to characterise H3N2 circulating strains are limited as a result of the receptor binding changes in the viruses which have occurred over recent years [26]. Such changes have altered the ability to use the traditional tools for antigenic characterisation of circulating strains using ferret post-infection antisera and HI antibody reactivity, as these tests rely on receptor binding to indicator red cells. The limited data available so far from characterisation of the antigenic profiles of circulating influenza virus strains using virus neutralisation does not reveal major antigenic variation between the various H3N2 genetic subgroups. However, it is notable that 3C.2a2 (subgroup 3) viruses have acquired the 3C.2a1 neuraminidase (NA) gene through reassortment, which may contribute to overall antigenicity of the emerging dominant H3N2 viruses, resulting in a poorer match to the 2017/18 vaccine virus [27]. As the neuraminidase content of vaccines is not standardised, and neuraminidase inhibiting (NI) antibodies in the population are not routinely measured, it is not possible to assess the contribution that this evolutionary change in the H3N2 virus will make to overall population immunity and susceptibility, and therefore VE. This is an area which requires further detailed and systematic study, including an understanding of the contribution of viral neuraminidase to overall viral fitness and immune escape. Natural virus evolution seems to have been exacerbated further by the egg-adaption of the vaccine virus; with the A(H3N2) age-specific susceptibility data from summer 2017, showing significantly lower sero-reactivity particularly in the elderly for the cell-derived A(H3N2) virus compared with the same age group for the egg-grown A(H3N2) virus, an observation which has been made elsewhere [7,28]. Tissue culture grown vaccine virus strains are considered more representative of the circulating virus strains which are recovered from human respiratory tract [15]. Detailed follow-up investigations of the molecular basis of the differences are required from this observation. However, it does highlight the potential value of seroprevalence surveys to identify possible susceptibility gaps in the population. Further work is required to disentangle the relative contribution of these different factors including yearly vaccination and immunosenescence. Though these results support the need for more effective interventions against A(H3N2), particularly for older people, where the burden of A(H3N2) is most notable [29], but also children, where the programme is intended to provide both direct protection to the children themselves, and by reducing their rates of infection, indirectly protect others in the population. WHO has recommended that the A(H3N2) component of the 2018/19 vaccine is updated to the A/Singapore/INFIMH-16–0019/2016 (H3N2)-like virus and the UK has preferentially recommended adjuvanted vaccine for the elderly in 2018/19, which is likely to enhance and increase the breadth of the immune response against A(H3N2). Finally, cell-based influenza vaccines, which avoid the issue of egg-adaption are now being used in North America and are now licensed for use in the UK in 2019/20. Indeed VE results from the 2017/18 season in the US suggest such vaccines offer significantly better protection compared with traditional egg-based, non-high dose vaccines in ≥ 65 year olds [30].

The present study does report significant LAIV4 effectiveness for children 2–17 years of age against influenza A(H1N1)pdm09. These results are particularly encouraging in the light of the temporary recommendation to not use LAIV4 in the US following the finding of reduced VE in 2015/16 [5]. The US results of no significant effectiveness against A(H1N1)pdm09 were at odds with those seen in several other countries that had used LAIV4 in 2015/16, including the UK [6,31], though all had noted lower effectiveness of LAIV4 against A(H1N1)pdm09 compared with IIV in 2015/16. One emerging hypothesis suggests this might relate to reduced replicative ability of the A(Bolivia/559/2013) (H1N1)pdm09 vaccine strain in LAIV4 [31], whereby this strain was updated to the A(Slovenia/2903/2015) (H1N1)pdm09 strain for the 2017/18 season. Recently presented results from the LAIV manufacturer in the US indicate more encouraging shedding and immunogenicity data of this new strain in young children compared with A/Bolivia/559/2013 [32]. The results in this study of good effectiveness against A(H1N1)pdm09 supports the ongoing roll-out of the UK paediatric influenza vaccine programme, although the reduced A(H3N2) effectiveness seen this season (due to likely egg-adaption) still needs to be addressed.

In summary, this work demonstrates a lack of significant effectiveness against A(H3N2) in all age groups possibly related to several factors most notably egg-adaption of the vaccine virus combined with the emergence of a new A(H3N2) subgroup. It is hoped that the impact of A(H3N2) will be mitigated by the updating of the A(H3N2) vaccine virus strain in 2018/19 and the availability of newly licensed adjuvanted, high-dose and cell-based vaccines in the UK. The result of lower effectiveness of inactivated trivalent vaccine against influenza B in adults seems most likely related to the B lineage mismatch this season. The introduction of quadrivalent influenza vaccine for adults in the UK in 2018/19 is intended to help to improve protection [33]. The VE results for both influenza B and A(H1N1)pdm09 in children are encouraging – though the poor performance against A(H3N2) this season will need to be monitored carefully.

## Supplementary Data

219000 Supplementary Material

## References

[1] Hakin B, Cosford P, Harvey F. The flu immunisation programme 2013/14– extension to children. London: Department of Health; 26 Jul 2013. Available from: https://www.gov.uk/government/uploads/system/uploads/attachment_data/file/225360/Children_s_flu_letter_2013.pdf

[2] Public Health England, Department of Health. Flu Plan Winter. 2017-18. London: NHS England; March 2017. Available from: https://assets.publishing.service.gov.uk/government/uploads/system/uploads/attachment_data/file/600532/annual_flu_plan_2017to2018.pdf

[3] World Health Organization (WHO). WHO recommended composition for 2017/18 Northern hemisphere influenza season. Geneva: WHO; 2 March 2017. Available from: https://www.who.int/influenza/vaccines/virus/recommendations/2017_18_north/en/

[4] Pebody R Warburton F Ellis J Andrews N Potts A Cottrell S Effectiveness of seasonal influenza vaccine for adults and children in preventing laboratory-confirmed influenza in primary care in the United Kingdom: 2015/16 end-of-season results. Euro Surveill. 2016;21(38):30348. 10.2807/1560-7917.ES.2016.21.38.30348 27684603 PMC5073201

[5] Grohskopf LA Sokolow LZ Broder KR Olsen SJ Karron RA Jernigan DB Prevention and Control of Seasonal Influenza with Vaccines. MMWR Recomm Rep. 2016;65(5):1-54. 10.15585/mmwr.rr6505a1 27560619

[6] Penttinen PM Friede MH Decreased effectiveness of the influenza A(H1N1)pdm09 strain in live attenuated influenza vaccines: an observational bias or a technical challenge? Euro Surveill. 2016;21(38):30350. 10.2807/1560-7917.ES.2016.21.38.30350 27684999 PMC5073203

[7] Skowronski DM Janjua NZ De Serres G Sabaiduc S Eshaghi A Dickinson JA Low 2012-13 influenza vaccine effectiveness associated with mutation in the egg-adapted H3N2 vaccine strain not antigenic drift in circulating viruses. PLoS One. 2014;9(3):e92153. 10.1371/journal.pone.0092153 24667168 PMC3965421

[8] Public Health England (PHE). Annual National Flu Report: Surveillance of influenza and other respiratory viruses in the UK: Winter 2017 to 2018. London: PHE; May 2018. Available from: https://assets.publishing.service.gov.uk/government/uploads/system/uploads/attachment_data/file/710483/Surveillance_of_influenza_and_other_respiratory_viruses_in_the_UK_2017_to_2018.pdf

[9] Fleming DM Andrews NJ Ellis JS Bermingham A Sebastianpillai P Elliot AJ Estimating influenza vaccine effectiveness using routinely collected laboratory data. J Epidemiol Community Health. 2010;64(12):1062-7. 10.1136/jech.2009.093450 19910645

[10] Gunson R Maclean A Davies E Bennett S Miller R Carman WF Development of a multiplex real-time RT-PCR that allows universal detection of influenza A viruses and simultaneous typing of influenza A/H1N1/2009 virus. J Virol Methods. 2010;163(2):258-61. 10.1016/j.jviromet.2009.10.006 19854220 PMC7173015

[11] Matrosovich M Matrosovich T Carr J Roberts NA Klenk HD Overexpression of the alpha-2,6-sialyltransferase in MDCK cells increases influenza virus sensitivity to neuraminidase inhibitors. J Virol. 2003;77(15):8418-25. 10.1128/JVI.77.15.8418-8425.2003 12857911 PMC165236

[12] Zambon M. Laboratory Diagnosis of Influenza. In: Nicholson K, Hay A, Webster RG, editors. Textbook of Influenza. Oxford: Blackwell Science; 1998. pp. 291-313].

[13] Tamura K Stecher G Peterson D Filipski A Kumar S MEGA6: Molecular Evolutionary Genetics Analysis version 6.0. Mol Biol Evol. 2013;30(12):2725-9. 10.1093/molbev/mst197 24132122 PMC3840312

[14] World Health Organization (WHO) Composition recommandée des vaccins antigrippaux pour la saison grippale 2017-2018 dans l’hémisphère Nord. [Recommended composition of influenza virus vaccines for use in the 2017–2018 northern hemisphere influenza season]. Wkly Epidemiol Rec. 2017;92(11):117-28. 28303704

[15] Zost SJ Parkhouse K Gumina ME Kim K Diaz Perez S Wilson PC Contemporary H3N2 influenza viruses have a glycosylation site that alters binding of antibodies elicited by egg-adapted vaccine strains. Proc Natl Acad Sci USA. 2017;114(47):12578-83. 10.1073/pnas.1712377114 29109276 PMC5703309

[16] Rondy M Kissling E Emborg HD Gherasim A Pebody R Trebbien R Interim 2017/18 influenza seasonal vaccine effectiveness: combined results from five European studies. Euro Surveill. 2018;23(9):1800086. 10.2807/1560-7917.ES.2018.23.9.18-00086 29510782 PMC5840921

[17] Flannery B Chung JR Belongia EA McLean HQ Gaglani M Murthy K Interim Estimates of 2017-18 Seasonal Influenza Vaccine Effectiveness - United States, February 2018. MMWR Morb Mortal Wkly Rep. 2018;67(6):180-5. 10.15585/mmwr.mm6706a2 29447141 PMC5815489

[18] Skowronski DM Chambers C De Serres G Sabaiduc S Winter AL Dickinson JA Vaccine effectiveness against lineage matched and mismatched influenza B viruses across 8 seasons in Canada, 2010-11 to 2017-18. Clin Infect Dis. 2018;68(10):1754-7. 10.1093/cid/ciy876 30312364 PMC6495010

[19] McLean HQ Thompson MG Sundaram ME Kieke BA Gaglani M Murthy K Influenza vaccine effectiveness in the United States during 2012-2013: variable protection by age and virus type. J Infect Dis. 2015;211(10):1529-40. 10.1093/infdis/jiu647 25406334 PMC4407759

[20] de Boer PT van Maanen BM Damm O Ultsch B Dolk FCK Crépey P A systematic review of the health economic consequences of quadrivalent influenza vaccination. Expert Rev Pharmacoecon Outcomes Res. 2017;17(3):249-65. 10.1080/14737167.2017.1343145 28613092

[21] Thorrington D van Leeuwen E Ramsay M Pebody R Baguelin M Cost-effectiveness analysis of quadrivalent seasonal influenza vaccines in England. BMC Med. 2017;15(1):166. 10.1186/s12916-017-0932-3 28882149 PMC5590113

[22] Hodgson D Baguelin M van Leeuwen E Panovska-Griffiths J Ramsay M Pebody R Effect of mass paediatric influenza vaccination on existing influenza vaccination programmes in England and Wales: a modelling and cost-effectiveness analysis. Lancet Public Health. 2017;2(2):e74-81. 10.1016/S2468-2667(16)30044-5 28299371 PMC5341148

[23] Pebody R Warburton F Ellis J Andrews N Potts A Cottrell S End-of-season influenza vaccine effectiveness in adults and children, United Kingdom, 2016/17. Euro Surveill. 2017;22(44): 1700306. 10.2807/1560-7917.ES.2017.22.44.17-00306 29113630 PMC5710133

[24] Skowronski DM Chambers C Sabaiduc S Dickinson JA Winter AL De Serres G Interim estimates of 2016/17 vaccine effectiveness against influenza A(H3N2), Canada, January 2017. Euro Surveill. 2017;22(6):30460. 10.2807/1560-7917.ES.2017.22.6.30460 28205503 PMC5316907

[25] Belongia EA Simpson MD King JP Sundaram ME Kelley NS Osterholm MT Variable influenza vaccine effectiveness by subtype: a systematic review and meta-analysis of test-negative design studies. Lancet Infect Dis. 2016;16(8):942-51. 10.1016/S1473-3099(16)00129-8 27061888

[26] Lin Y Wharton SA Whittaker L Dai M Ermetal B Lo J The characteristics and antigenic properties of recently emerged subclade 3C.3a and 3C.2a human influenza A(H3N2) viruses passaged in MDCK cells. Influenza Other Respir Viruses. 2017;11(3):263-74. 10.1111/irv.12447 28164446 PMC5410720

[27] World Health Organization (WHO). Recommended composition of influenza virus vaccines for use in the 2018 southern hemisphere influenza season. Geneva: WHO; 28 September 2017. Available from: https://www.who.int/influenza/vaccines/virus/recommendations/201709_recommendation.pdf?ua=1

[28] Zost SJ Parkhouse K Gumina ME Kim K Diaz Perez S Wilson PC Contemporary H3N2 influenza viruses have a glycosylation site that alters binding of antibodies elicited by egg-adapted vaccine strains. Proc Natl Acad Sci USA. 2017;114(47):12578-83. 10.1073/pnas.1712377114 29109276 PMC5703309

[29] Shay DK Chillarige Y Kelman J Forshee RA Foppa IM Wernecke M Comparative Effectiveness of High-Dose Versus Standard-Dose Influenza Vaccines Among US Medicare Beneficiaries in Preventing Postinfluenza Deaths During 2012-2013 and 2013-2014. J Infect Dis. 2017;215(4):510-7. 10.1093/infdis/jiw641 28329311

[30] Izurieta HS Chillarige Y Kelman J Wei Y Lu Y Xu W Relative effectiveness of cell-cultured and egg-based influenza vaccines among the U.S. elderly, 2017-18. J Infect Dis. 2018. 10.1093/infdis/jiy716 30561688

[31] Ambrose CS Bright H Mallory R Letter to the editor: Potential causes of the decreased effectiveness of the influenza A(H1N1)pdm09 strain in live attenuated influenza vaccines. Euro Surveill. 2016;21(45):30394. 10.2807/1560-7917.ES.2016.21.45.30394 27918259 PMC5144940

[32] Mallory R. Results of Randomized Trial of a New H1N1 LAIV Strain in US Children. Presentation to ACIP 21 Feb 2018. page 53. Available from: https://www.cdc.gov/vaccines/acip/meetings/downloads/min-archive/min-2018-02-508.pdf

[33] Public Health England, Department of Health. Annual Flu Letter. 2018/19. London: NHS; 26 March 2018. Available from: https://assets.publishing.service.gov.uk/government/uploads/system/uploads/attachment_data/file/694779/Annual_national_flu_programme_2018-2019.pdf

