# Supplementary material for: End of season influenza vaccine effectiveness in adults and children in the United Kingdom in 2017/18
Source: Euro Surveill. 2019 Aug 1;24(31):1800488. doi: 10.2807/1560-7917.ES.2019.24.31.1800488 (PMC6685099; doi:10.2807/1560-7917.ES.2019.24.31.1800488)
Supplement: Supplementary Material [file 18-00488_PEBODY_influenza_Supplement.pdf]

Supplement– Details of influenza A/H3N2 haemagglutinin sequences obtained from GISAID used in the phylogenetic analysis.

This supplementary material is hosted by Eurosurveillance as supporting information alongside the article *End of season influenza vaccine effectiveness in adults and children in the United Kingdom in 2017/18* on behalf of the authors who remain responsible for the accuracy and appropriateness of the content. The same standards for ethics, copyright, attributions and permissions as for the article apply. Eurosurveillance is not responsible for the maintenance of any links or email addresses provided therein.

CONFIDENTIAL

Virus isolate	Segment ID/Accession number	Country	Collection date (year-month-day)	Originating laboratory	Submitting laboratory
A/Samara/73/2013	EPI460558	Russian Federation	2013-Mar-12	WHO National Influenza Centre, Saint Petersburg, Russian Federation	National Institute for Medical Research, London, UK
A/Switzerland/9715293/2013	EPI530687	Switzerland	2013-Dec-06	Hopital Cantonal Universitaire de Geneves, Switzerland	National Institute for Medical Research, London, UK
A/Hong Kong/4801/2014	EPI539576	Hong Kong (SAR)	2014-Feb-26	Government Virus Unit, Hong Kong (SAR)	National Institute for Medical Research, London, UK
A/New Caledonia/71/2014	EPI551570	New Caledonia	2014-Aug-13	Institut Pasteur New Caledonia, New Caledonia	WHO Collaborating Centre for Reference and Research on Influenza, Melbourne, Australia
A/Texas/50/2012	EPI556816	United States	2012-Apr-15	Texas Department of State Health Services-Laboratory Services, Austin, USA	Centers for Disease Control and Prevention, Atlanta, USA
A/Bolzano/7/2016	EPI773595	Italy	2016-Mar-15	Istituto Superiore di Sanità, Roma, Italy	Crick Worldwide Influenza Centre, London, UK
A/Scotland/63440583/2016	EPI831436	United Kingdom	2016-Aug-25	Gart-Naval General Hospital, Glasgow, Scotland, UK	Microbiology Services Colindale, Public Health England, London, UK
A/Singapore/INFIMH-16-0019/2016	EPI1047604	Singapore	2016-Jun-14	WHO Collaborating Centre for Reference and Research on Influenza, Melbourne, Australia	Centers for Disease Control and Prevention, Atlanta, USA
B/Phuket/3073/2013	EPI540675	Australia	2013-Nov-21	WHO Collaborating Centre for Reference and Research on Influenza, Melbourne, Australia	Crick Worldwide Influenza Centre, London, UK
B/Massachusetts/02/2012	EPI438406	United States	2012-Jan-01	New York Medical College, New York, USA	Centers for Disease Control and Prevention, Atlanta, USA
B/Wisconsin/01/2010	EPI271545	United States	2010-Feb-20	Wisconsin State Laboratory of Hygiene, Madison, USA	Centers for Disease Control and Prevention, Atlanta, USA
B/Malaysia/2506/2004	EPI175755	Malaysia	2004-Jan-01		Other Database Import (GenBank CY038287)
B/Brisbane/60/2008	EPI172555	Australia	2008-Aug-04	Centers for Disease Control and Prevention, Atlanta, USA	Centers for Disease Control and Prevention, Atlanta, USA

B/Bangladesh/3333/2007	EPI156050	Bangladesh	2007-Aug-18	Centers for Disease Control and Prevention, Atlanta, USA	Centers for Disease Control and Prevention, Atlanta, USA
B/Estonia/77391/2013	EPI467120	Estonia	2013-Apr-08	Health Protection Inspectorate, Tallin, Estonia	Crick Worldwide Influenza Centre, London, UK
B/Odessa/3886/2010	EPI902450	Ukraine	2010-Mar-19	Ministry of Health of Ukraine, Kiev, Ukraine	Crick Worldwide Influenza Centre, London, UK
B/Hong Kong/330/2001	EPI20103	China	2001		Other Database Import (GenBank AY504610)
B/Yamagata/16/88	EPI51820	Japan	1988		Other Database Import (GenBank AY018765)
B/Victoria/2/87	EPI130089	Australia	1987		Other Database Import (GenBank M58428)

GISAID: Global Initiative on Sharing All Influenza Data; SAR: Special Administrative Regions of the People's Republic of China; UK: United Kingdom; USA: United States of America; WHO: World Health Organization.

CONFIDENTIAL

CONFIDENTIAL
